# A Functional Polymorphism in Renalase (Glu37Asp) Is Associated with Cardiac Hypertrophy, Dysfunction, and Ischemia: Data from the Heart and Soul Study

**DOI:** 10.1371/journal.pone.0013496

**Published:** 2010-10-20

**Authors:** Ramin Farzaneh-Far, Gary V. Desir, Beeya Na, Nelson B. Schiller, Mary A. Whooley

**Affiliations:** 1 Division of Cardiology, San Francisco General Hospital, San Francisco, California, United States of America; 2 Department of Medicine, University of California San Francisco, San Francisco, California, United States of America; 3 Department of Internal Medicine, Yale School of Medicine, New Haven, Connecticut, United States of America; 4 Veterans Affairs Medical Center, San Francisco, California, United States of America; 5 Department of Epidemiology and Biostatistics, University of California San Francisco, San Francisco, California, United States of America; Maastricht University, Netherlands

## Abstract

**Background:**

Renalase is a soluble enzyme that metabolizes circulating catecholamines. A common missense polymorphism in the flavin-adenine dinucleotide-binding domain of human renalase (Glu37Asp) has recently been described. The association of this polymorphism with cardiac structure, function, and ischemia has not previously been reported.

**Methods:**

We genotyped the rs2296545 single-nucleotide polymorphism (Glu37Asp) in 590 Caucasian individuals and performed resting and stress echocardiography. Logistic regression was used to examine the associations of the Glu37Asp polymorphism (C allele) with cardiac hypertrophy (LV mass>100 g/m2), systolic dysfunction (LVEF<50%), diastolic dysfunction, poor treadmill exercise capacity (METS<5) and inducible ischemia.

**Results:**

Compared with the 406 participants who had GG or CG genotypes, the 184 participants with the CC genotype had increased odds of left ventricular hypertrophy (OR = 1.43; 95% CI 0.99–2.06), systolic dysfunction (OR = 1.72; 95% CI 1.01–2.94), diastolic dysfunction (OR = 1.75; 95% CI 1.05–2.93), poor exercise capacity (OR = 1.61; 95% CI 1.05–2.47), and inducible ischemia (OR = 1.49, 95% CI 0.99–2.24). The Glu37Asp (CC genotype) caused a 24-fold decrease in affinity for NADH and a 2.3-fold reduction in maximal renalase enzymatic activity.

**Conclusions:**

A functional missense polymorphism in renalase (Glu37Asp) is associated with cardiac hypertrophy, ventricular dysfunction, poor exercise capacity, and inducible ischemia in persons with stable coronary artery disease. Further studies investigating the therapeutic implications of this polymorphism should be considered.

## Introduction

The kidney, in addition to maintaining fluid and electrolyte homeostasis, performs essential endocrine functions [1]. Patients with end-stage renal disease are at high risk for cardiovascular events, even when provided optimal renal replacement therapy [2], [3]. It has been suggested that failure to replicate the endocrine functions of the kidney may contribute to this risk, in association with heightened sympathetic tone [4], [5], [6]. We recently identified renalase, a flavin adenine dinucleotide-dependent amine oxidase that is secreted into the blood by the kidney, metabolizes circulating catecholamines, and is deficient in chronic kidney disease [7]. Excess catecholamines promote the activity, secretion, and synthesis of renalase, providing a novel pathway of negative-feedback homeostatic control [8]. In rodents, parenteral administration of renalase lowers blood pressure, heart rate, and cardiac contractility [9]. During cardiac ischemia in rats, infusion of recombinant renalase reduces myocardial infarct size whereas neonatal nephrectomy leads to elevated sympathetic nervous system activity, renalase deficiency, and cardiac hypertrophy [10], [11].

Human renalase is encoded by a 311Kbp gene with 10 exons located on chromosome 10q23.33. The renalase protein is well conserved with orthologs present in chimpanzees (95% amino acid identity) and cyanobacteria (23% identity). The major isoform of renalase contains 342 amino acids comprising a signal peptide (amino acids 1–17), a flavin-adenine dinucleotide (FAD) binding domain (amino acids 4–45), and a monoamine oxidase domain (amino acids 75–342). Evidence exists for at least four alternatively-spliced isoforms of renalase [12]. The most common isoform (renalase1) is encoded by exons 1–4, 6–7, and 9. It is the predominant human renalase protein detectable in plasma, kidney, heart, skeletal muscle, and liver. The functional significance of the spliced isoforms is not known. A common missense polymorphism in the flavin-adenine dinucleotide-binding domain of human renalase (Glu37Asp) has recently been described. This is the only reported common coding single-nucleotide polymorphism in the renalase gene, and was recently found to be associated with essential hypertension [13].

Whether common genetic variation at this locus affects cardiac structure, function, and ischemia in humans is not known. We therefore genotyped the (Glu37Asp) polymorphism and evaluated its association with cardiovascular phenotypes in a large cohort of individuals with stable coronary artery disease.

## Methods

### Participants

The Heart and Soul Study is a prospective cohort study investigating the influence of psychosocial factors on cardiovascular events in outpatients with stable coronary artery disease. The enrollment process for the Heart and Soul Study has been previously described [14]. Eligible participants were recruited from outpatient clinics in the San Francisco Bay Area if they met at least one of the following inclusion criteria: 1) history of myocardial infarction, 2) angiographic evidence of at least 50% stenosis by area in at least 1 coronary artery, 3) evidence of exercise-induced ischemia by treadmill electrocardiogram or stress nuclear perfusion imaging, or 4) history of coronary revascularization. Individuals were excluded if they had a history of myocardial infarction in the past 6 months, deemed themselves unable to walk 1 block, or if they were planning to move out of the local area within 3 years. There was no exclusion on the basis of kidney disease.

The study protocol was approved by the following Institutional Review Boards: the University of California San Francisco Committee on Human Research, the Research and Development Committee at the San Francisco VA Medical Center, the Medical Human Subjects Committee at Stanford University, the Human Subjects Committee at the VA Palo Alto Health Care System, and the Data governance Board of the Community Health Network of San Francisco. All participants provided written informed consent. Between September 2000 and December 2002, a total of 1024 participants enrolled in the study. Of these, DNA samples were available for analysis in 982 individuals, including 590 of Caucasian ethnicity. There were no demographic differences between individuals who did and those who did not provide DNA samples for analysis.

### Genotyping

Prior to the study appointment, participants completed an overnight fast except for taking their regularly prescribed medications. Fasting venous blood samples were drawn into chilled ethylene-diamine-tetra-acetic acid tubes, and peripheral blood leukocytes were stored at −70° Celsius. Samples were later thawed, and genomic DNA was extracted using a salt modification method (Gentra Systems). The ABI PRISM 7900HT Sequence Detection System was used to perform genotyping of the rs2296545 single nucleotide polymorphism using the 5′nuclease allelic discrimination assay (Taqman Assay, Applied Biosystems, Foster City, USA). The assay kit included the forward and reverse target-specific polymerase chain reaction primers, and the Taqman MGB probes labeled with dyes DFAM and VIC. The total volume of the sequencing reaction was 5-ul containing 2.5ul of Taqman Universal PCR Master Mix, 0.083ul of 40X Taqman MGB Assay Mix, 1.417ul of ddH2O, and 1ul (5 ng) of genomic DNA. Investigators blinded to the clinical data performed the genotyping assays.

### Cardiac Structure, Function, and Ischemia

All participants underwent complete resting 2-dimensional echocardiography and Doppler examination using an Acuson Sequoia ultrasound system (Siemens Medical Solutions, Mountain View, CA) with a 3.5-MHz transducer. Standard parasternal short-axis and apical 2 – and 4-chamber views were obtained and planimetered to determine end-diastolic and end-systolic volumes. The left ventricular ejection fraction (LVEF) was calculated as (end diastolic volume – end systolic volume)/end diastolic volume. Systolic dysfunction was defined as LVEF<50%. Diastolic dysfunction was defined as the presence of one of the following: pseudonormal transmitral inflow pattern defined as a ratio of peak mitral early diastolic to atrial contraction velocity (E/A) of 0.75<E/A<1.5 with diastolic dominant pulmonary vein flow; restrictive filling defined as an E/A of 1.5 or greater with diastolic dominant pulmonary vein flow [15]. Left ventricular mass was calculated using a truncated ellipsoid equation as previously validated [16]. Left ventricular hypertrophy was defined as LV mass index>100 g/m^2^.

All participants then underwent a symptom-limited graded exercise treadmill test according to a standard Bruce protocol. To achieve maximal heart rate, participants who were unable to continue the standard Bruce protocol were switched to lower manual settings on the treadmill and encouraged to exercise for as long as possible. Maximal exercise capacity in metabolic equivalents (METS) was determined at peak exercise. Poor exercise capacity was defined as<5 METS at peak exercise [17]. Inducible ischemia was defined as the presence of one or more new wall motion abnormalities occurring with exercise. The post-exercise wall motion score was calculated according to the American Society of Echocardiography guidelines [18]. Resting and stress echocardiograms were read by a single, experienced reader (N.B.S.) who was blinded to genotype and outcome data.

### Other Patient Characteristics

Baseline demographics, age, sex, and ethnicity were determined by self-reported questionnaire. Cardiovascular co-morbidities including hypertension, diabetes, hyperlipidemia, prior myocardial infarction, prior congestive heart failure, prior stroke, smoking status, and prior revascularization were determined by self-report. Recumbent blood pressure was measured after 5 minutes at rest. Participants were weighed and measured without shoes. Body Mass Index (BMI) was calculated as the ratio of mass (kg) to the square of height (m^2^). All participants were instructed to bring their medication bottles to the study appointment where study personnel recorded all current medications. Medications were categorized using Epocrates Rx (San Mateo, CA), an electronic reference database that contains 3300 drugs classified by both generic and commercial product names. Fasting serum chemistry samples were used to measure triglycerides, HDL cholesterol, LDL cholesterol. Serum creatinine was measured by the rate Jaffe method. The intra-individual coefficient of variation was 2%. Estimated glomerular filtration rate (GFR) was calculated by the abbreviated (4-variable) Modification of Diet and Renal Disease Study formula as follows: estimated GFR = 186x(serum creatinine^−1.154^)x(age^−0.203^)x(0.742 if female)x(1.21 if black) [19].

### Synthesis and functional analysis of the renalase variants Glu37 and Asp37

Untagged,recombinant renalase variants, Glu37 and Asp37, were generated by cloning the respective coding region into the Ned1/Xho1 sites of the Pet27b+ vector (Novagen, Madison, WI). E. coli BL21 were transformed and grown at 37°C for 16 hours with 0.1 µM flavin adenine dinucleotide (FAD). Isopropyl β-D-1-thiogalactopyranoside (IPTG) was added for the last 3.5 hours of culture. Recombinant renalase was purified from inclusion bodies and refolded as previously described [7].

Since renalase is a NADH/FAD dependent oxidoreductase, the enzymatic activity the each variant was assessed by measuring the rate of reduction of 2-(4-iodophenyl)-3-(4-nitrophenyl)-5-(2,4-disulfophenyl)-2H tetrazolium monosodium salt (WST-1) as a function of NADH concentration. Reduction of WST-1 results in the formation of a yellow, water-soluble formazan product, which can be quantified by measuring absorbance at 450 nm [20], [21]. The assay buffer contained 50 mM potassium phosphate, 0.5 mM WST-1 (Dojindo Molecular Technologies, Inc), 1 mM EDTA, pH 7.5. NADH concentration was varied from 0 to 2 mM. The reactions were initiated by adding 20 µg of 37Glu, or 37Asp, or bovine serum albumin (BSA) as a negative control, in a total volume of 200 µl, in 96-well plate cuvettes (0.6 cm path length). Changes in absorbance at 37°C were recorded every minute for 15 minutes. The amount of WST-1 reduced by renalase was calculated from the increase in absorbance at 450 nm, and using a molar extinction coefficient of 37,000 M^−1^ cm-. Background correction was achieved by subtracting the changes in absorbance obtained with BSA. To estimate kinetics parameters, initial velocity was plotted against NADH concentration, and the data were fitted to the Michaelis-Menten equation using non-linear regression (GraphPad Prism, GraphPad Software, Inc.).

### Statistical Analysis

Differences in baseline characteristics between participants with distinct rs2296545 genotypes (GG, GC, and CC) were compared with the use of analysis of variance for continuous variables and the chi-squared test for dichotomous variables. We then examined differences in cardiac phenotype by genotype using generalized linear models for continuous measures and logistic regression for dichotomous measures of cardiac function. Participants with GG and GC genotypes were combined for multivariable analyses because they had similar cardiac function in unadjusted analyses. Multivariable adjustment was made for age, gender, BMI, systolic blood pressure, diastolic blood pressure, and estimated GFR. Covariates were selected on their potential for confounding or mediating effects. Model assumptions were checked by visual inspection of directed acyclic graphs [22]. To explore the possibility of effect modification by age, we tested for statistical interactions between renalase genotype and measures of cardiovascular structure and function in the multivariable adjusted models.

Statistical analysis was performed using SAS software version 9.1 (SAS Institute Inc, Cary, NC). The authors take responsibility for the integrity of the data. All authors had full access to the data, except N.B.S. who was blinded to the genotype data. All authors have read and agree to the manuscript as written.

## Results

We genotyped 590 Caucasian subjects in the Heart and Soul Study for the rs2296545 (Glu37Asp) single-nucleotide polymorphism. The genotype distribution was GG (107), CG (299), and CC (184). The observed frequencies of the G and C alleles were 0.43 and 0.57 respectively. These findings were consistent with Hardy-Weinberg equilibrium (p = 0.99). In a Han Chinese population, the observed frequencies of the G and C alleles were 0.39 and 0.61 in hypertensive individuals, and 0.44 and 0.56 in controls respectively [13]. The baseline characteristics of the study population categorized by genotype at rs2296545 are shown in **Table 1**. There were no significant associations between rs1333049 genotype and baseline demographics, medical history, smoking, medication use, blood pressure, or lipids. In bivariate analysis, GG homozygosity was associated with a prior history of revascularization (p = 0.04).

**Table 1:** Baseline characteristics of participants by genotype at rs2296545.

Variable	GG(Glu/Glu)N = 107	CG(Glu/Asp)N = 299	CC(Asp/Asp)N = 184	P value(ANOVA or chi^2^)
Age	67±10	68±11	68±12	.89
Male (%)	89(83)	248(83)	163(89)	.22
BMI (kg/m2)	28.4±4.6	28.5±5.4	28.7±5.1	.88
Medical History (%)				
Hypertension	65(61)	196(66)	125(68)	.46
Myocardial infarction	60(56)	166(56)	103(56)	1.0
CHF	16(15)	46(15)	43(24)	.06
Stroke	10(9)	38(13)	28(15)	.35
Diabetes	22(21)	56(19)	43(23)	.48
Revascularization	80(75)	184(62)	116(63)	.04
Current Smoking	18(17)	45(15)	37(20)	.35
Medication Use (%)				
Statin	71(66)	200(67)	115(63)	.60
Beta-blocker	59(55)	174(58)	103(56)	.82
Aspirin	86(80)	235(79)	139(76)	.59
ACE-I/ARB	57(53)	151(51)	99(54)	.75
Systolic BP (mmHg)	130.3±22.5	131.1±20.1	131.6±20.0	.87
Diastolic BP (mmHg)	72.9±11.1	73.3±10.7	73.2±11.3	.94
Triglycerides (mg/dL)	140.8±96.9	149.2±150.2	144.0±130.7	.83
LDL-Cholesterol (mg/dL)	97.5±30.8	104.8±34.9	102.1±32.6	.16
HDL-Cholesterol (mg/dL)	43.6±12.0	45.4±15.1	46.1±15.7	.36
Creatinine (mg/dL)	1.1±0.3	1.1±0.3	1.1±0.5	.09
Estimated GFR (ml/min)	79.0±25.2	81.4±26.8	78.4±29.7	.49

Parameters of cardiovascular structure, function, and ischemia categorized by genotype at rs2296545 are shown in **Table 2**. The CC homozygous genotype was associated with lower ejection fraction (p = 0.01), higher left ventricular mass index (p = 0.008), lower exercise capacity (p = 0.002), and more diastolic dysfunction (p = 0.05). There was a trend towards more inducible ischemia in the CC group (p = 0.09). After multivariable adjustment the CC homozygous genotype remained associated with lower ejection fraction (p = 0.01), higher left ventricular mass index (p = 0.01), and lower exercise capacity (p = 0.003) **(** **Table 3** **)**.

**Table 2:** Parameters of cardiovascular structure and function by genotype at rs2296545 (amino acid 37).

	Genotype			
Variable	GG(Glu/Glu)N = 107	GC(Glu/Asp)N = 299	CC(Asp/Asp)N = 184	P value for trend
LVEF (%)	60.9±9.2	62.6±9.0	59.8±11.4	0.01
LV Mass Index (g/m2)	96.6±22.6	94.3±24.7	101.7±26.5	0.008
Exercise Capacity (METS)	7.8±3.9	7.7±3.4	6.6±3.3	0.002
Diastolic Dysfunction (%)	14(15)	28(11)	32(19)	0.05
Inducible Ischemia (%)	21(21)	73(26)	57(33)	0.09

**Table 3:** Association of CC (vs. GG or CG) genotype at rs2296545 with continuous measures of cardiovascular structure and function.

	Mean (±SE)		
	CC(Asp/Asp)N = 184	GC or GG(Glu/Asp or Glu/Glu)N = 406	P value
**LVEF (%)**			
Adjusted for age and gender	60.8(0.85)	63.0(0.62)	0.01
Multivariable adjusted *	60.7(0.86)	63.0(0.62)	0.01
**LV Mass Index (g/m2)**			
Adjusted for age and gender	96.3(2.11)	90.3(1.53)	.006
Multivariable adjusted *	95.4(2.10)	90.0(1.53)	.01
**Exercise Capacity (METS)**			
Adjusted for age and gender	6.0(0.28)	7.2(0.21)	.0001
Multivariable adjusted *	6.2(0.27)	7.2(0.20)	.0003

Adjusted for age, gender, body mass index, systolic blood pressure, diastolic blood pressure and estimated glomerular filtration rate.

**Figure 1:**
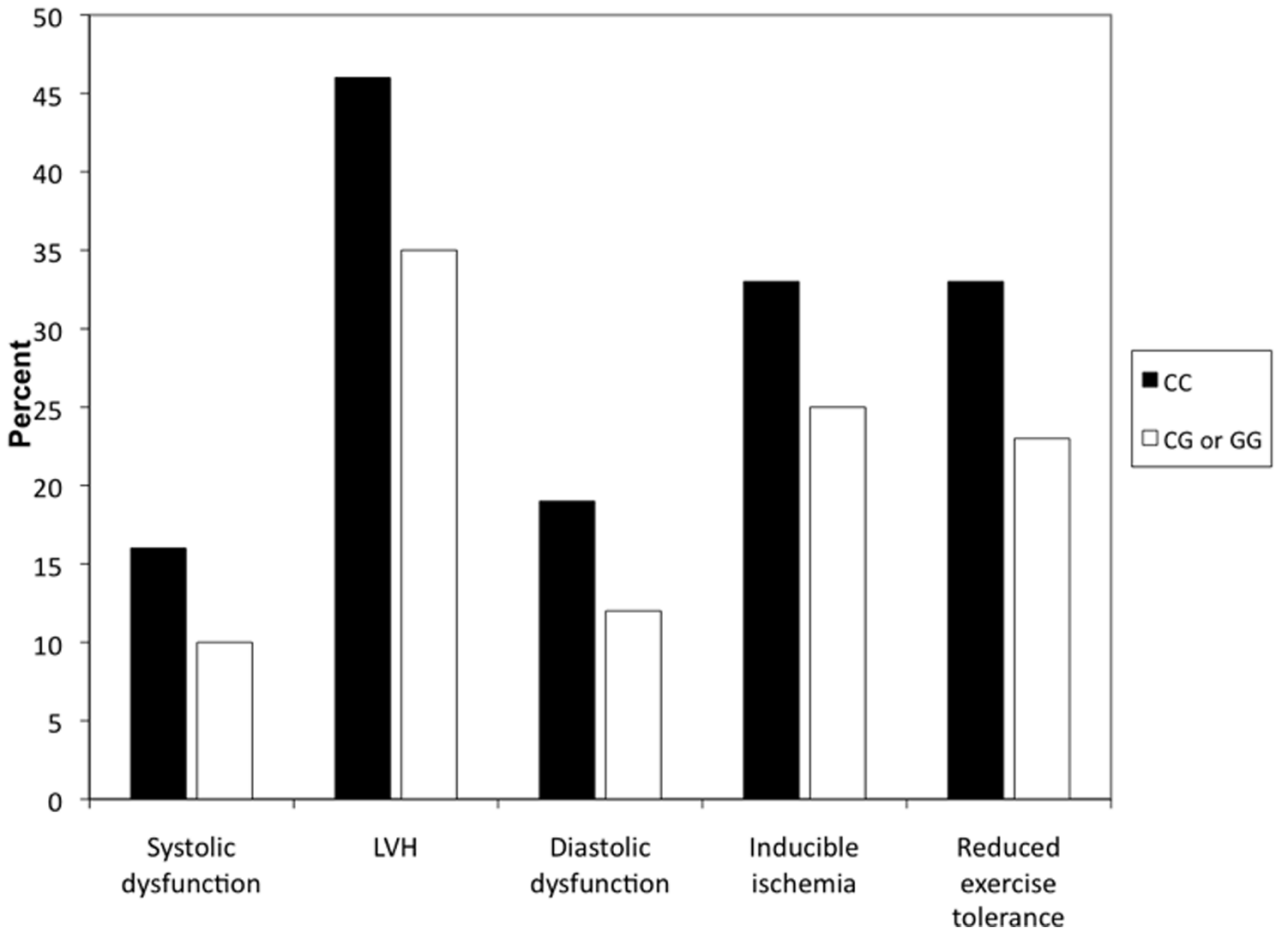
Proportion of participants with abnormal cardiovascular phenotype classified by genotype at rs2296545 (p<0.05 for all comparisons).

In logistic regression models adjusted for age, demographics, BMI, systolic and diastolic blood pressure, and estimated GFR, the CC genotype was associated with left ventricular hypertrophy (OR = 1.43, 95% CI 0.99–2.06; p = 0.06), systolic dysfunction (OR = 1.72, 95% CI 1.01–2.94; p = 0.05), diastolic dysfunction (OR = 1.75, 95% CI 1.05–2.93; p = 0.03), poor exercise capacity (OR = 1.61, 95% CI 1.05–2.47; p = 0.03), and inducible ischemia (OR = 1.49, 95% CI 0.99–2.24; p = 0.06) **(** **Table 4** **).** The proportion of participants with abnormal cardiovascular phenotype classified by genotype at rs2296545 is shown in **Figure 1** **.** We found no evidence that the association of renalase genotype with cardiovascular structure and function was modified by age (all p values for interaction>0.2).

**Table 4:** Association of CC (vs. GG or CG) genotype at rs2296545 with dichotomous measures of cardiovascular structure and function.

OUTCOME	Adjusted forage and genderOR (95% CI)	p	Multivariable adjusted*OR (95% CI)	p
Systolic Dysfunction(LVEF<50%)	1.64 (0.97–2.78)	.06	1.72 (1.01–2.94)	.05
Left Ventricular Hypertrophy (LVMI>100 g/m2)	1.48 (1.03–2.12)	.04	1.43 (0.99–2.06)	.06
Diastolic Dysfunction	1.76 (1.07–2.92)	.03	1.75 (1.05–2.93)	.03
Inducible Ischemia	1.48 (0.99–2.22)	.06	1.49 (0.99–2.24)	.06
Poor Exercise Capacity(<5 METS)	1.67 (1.09–2.54)	.02	1.61 (1.05–2.47)	.03

*Adjusted for age, gender, body mass index, systolic blood pressure, diastolic blood pressure and estimated glomerular filtration rate.

**Figure 2:**
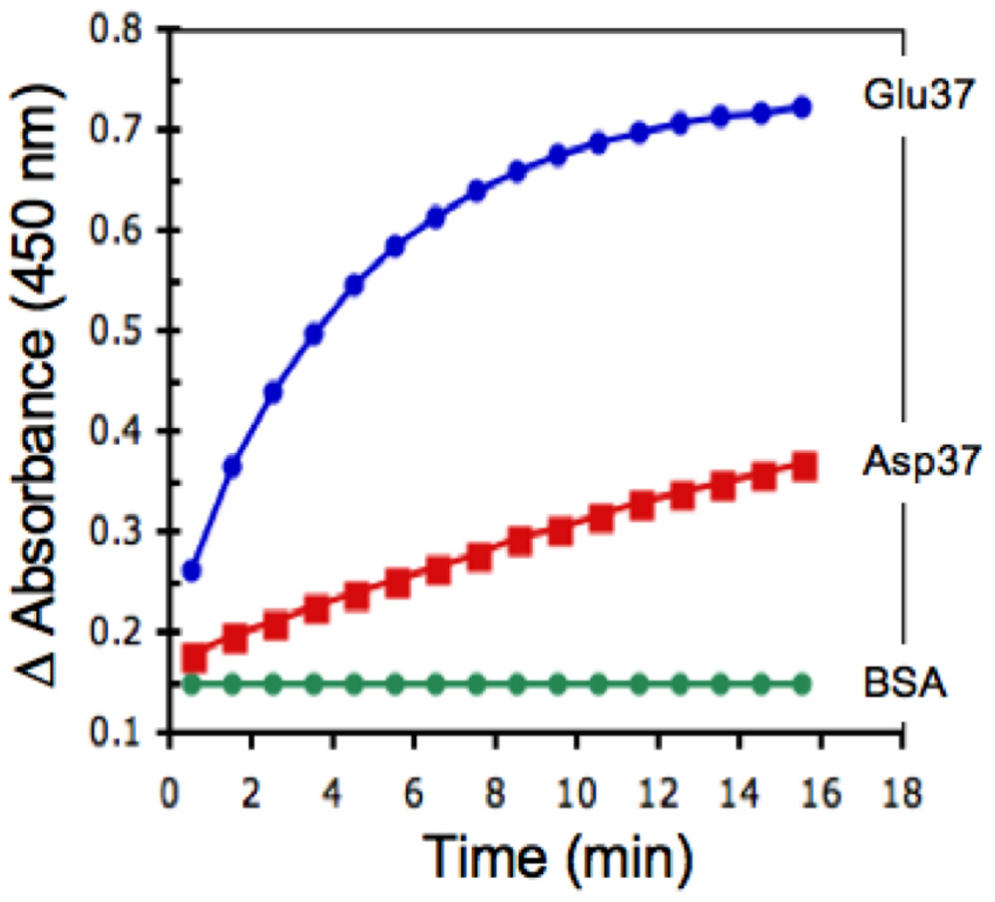
Enzymatic activity and kinetics parameters of renalase variants. Change in absorbance reflects the rate of WST-1 reduction, which is index of NADH oxidation; Glu37: glutamic acid at amino acid 37; Asp37: aspartic acid at amino acid 37; BSA, bovine serum albumin; raw data shown for a single experiment.

**Figure 3:**
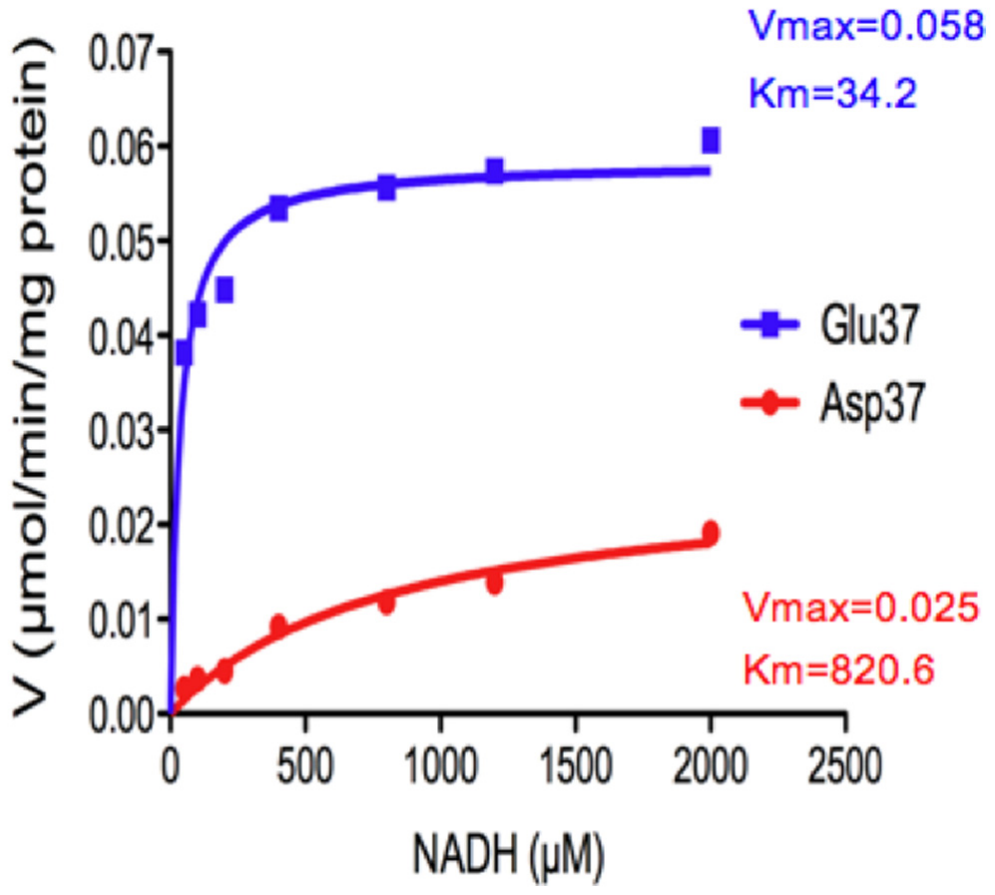
Calculated kinetics parameters: Glu37 =  34.1±4.0 µM; Asp37 = 820±115.1 µM, p<0.000001, n = 3; Vmax : Glu37 =  58.3±1.1 nmol/min/mg; Asp37 = 25.4±1.6 nmol/min/mg, p<0.0001, n = 3; Km: substrate concentration required to reach ½ maximal velocity; Vmax: maximal velocity; V =  velocity.

Renalase is a FAD dependent oxidoreductase, which uses NADH as a cofactor to reduce its FAD moiety. In the presence of oxygen the reduced renalase-FADH complex metabolizes subtrates such as catecholamines. Renalase has intrinsic NADH oxidase activity since, in the absence of substrates, it can convert NADH to NAD+ and generate superoxide anion as a byproduct. To test if the Glu37Asp polymorphism had any detectable functional consequence, we compared the NADH oxidase activity of the Glu37 and Asp37 variants of the recombinant renalase by measuring the rate of reduction, of the electron acceptor WST-1, as a function of NADH concentration. At all NADH concentrations tested (up to 2 mM,) Glu37 metabolized NADH at a significantly faster rate than Asp37. **Figure 2** depicts the results of a representative experiment with a NADH concentration of 400 µM. Kinetics parameters were determined by fitting (non linear regression) initial rate data to the Michaelis-Menten equation. The Glu37Asp mutation caused a 24 fold increase in Km (Glu37 =  34.1±4.0 µM; Asp37 = 820±115.1 µM, p<0.000001, n = 3), and a 2.3 fold reduction in Vmax (Glu37 =  58.3±1.1 nmol/min/mg; Asp37 = 25.4±1.6 nmol/min/mg, p<0.0001, n = 3).; **Figure 3** **.**

## Discussion

The recent discovery of renalase has elucidated a novel pathway for the homeostatic control of circulating catecholamines. Data from the International HapMap Consortium has identified a single common non-synonymous single-nucleotide polymorphism in the human renalase gene that results in an aspartic acid to glutamic acid substitution at codon 37 (Glu37Asp) in the flavin-adenine dinucleotide-binding site. In the present study of 590 persons with stable coronary artery disease, we report an association of CC (Asp/Asp) homozygosity at codon 37 with cardiac hypertrophy, dysfunction, and ischemia. Although this is a conservative substitution (Glu37Asp), it does occur in a critical region of the protein, namely the FAD binding domain, And it is, therefore, not surprising that compared to Glu37, Asp37 has a significantly lower affinity for NADH and a reduced maximal velocity. These findings raise the intriguing possibility that decreased activity of the enzyme renalase may contribute to cardiovascular disease.

At baseline, renalase activity in blood mirrors sympathetic tone [8]. Brief surges in catecholamine levels increase the activity, secretion, and synthesis of renalase resulting in accelerated degradation of catecholamines via a negative feedback mechanism. In animal models, catecholamine regulation by renalase demonstrates significant hemodynamic effects that mimic nonselective beta-adrenoceptor blockade: reduced heart rate, blood pressure, and cardiac contractility [7]. However, studies examining the *in vivo* relevance of renalase in humans are lacking. In a large case-control study of a northern Han Chinese population, Zhao et al found an association of the (Glu37Asp) polymorphism with essential hypertension (odds ratio 1.61; 95% CI 1.26–2.04; p = 0.0002) [13]. By contrast we found no significant association of the (Glu37Asp) polymorphism with either systolic or diastolic blood pressure, possibly because of more aggressive blood pressure control in patients with coronary artery disease. This discrepancy may reflect differences in study populations (essential hypertension versus stable coronary artery disease) and commensurate differences in medication use.

Accumulating evidence implicates excessive circulating catecholamines in the pathogenesis of left ventricular hypertrophy, a powerful independent risk factor for cardiovascular death and morbidity [23], [24], [25]. The observation that left ventricular hypertrophy is common in patients with end-stage renal disease has suggested a link between kidney function and cardiac hypertrophy [26], [27], [28]. Ghosh et al recently demonstrated that neonatal nephrectomy in rats results in cardiac hypertrophy associated with decreased norepinephrine metabolism [11]. Notably, renalase protein expression in cardiac tissue from nephrectomized rats was significantly lower than in controls. In the present study, we found an association of Asp/Asp homozygosity at codon 37 with increased left ventricular mass index (multivariable adjusted effect size = +5.4 g/m^2^). This supports a mechanistic link between the renalase-catecholamine axis and left ventricular hypertrophy, and extends this finding to humans. Further studies in animal models are warranted to investigate the relative contributions of renalase secreted from the kidney and the heart to the inhibition of cardiac hypertrophy.

The association of the Glu37Asp polymorphism with cardiac dysfunction has not previously been reported. Renalase infusion in rats results in an increase in stroke volume, which may reflect a compensatory response to decreased heart rate. However, since renalase also reduces cardiac contractility, an alternative explanation is that renalase-induced metabolism of circulating catecholamines increases cardiac lusitropy and optimizes diastolic-systolic coupling. We found essentially similar odds ratios for both systolic and diastolic dysfunction associated with Asp/Asp homozygosity in multivariable adjusted models further suggesting an influence of the renalase-catecholamine axis on both aspects of cardiac function. Detailed hemodynamic studies examining the effects of renalase administration on diastolic function would be of interest in this regard.

Cardiac ischemia, whether acute or chronic, is associated with a significant elevation in circulating catecholamines that promote and exacerbate myocardial infarction [29]. Pharmacologic blockade of beta adrenoceptors has therefore become an essential component of medical therapy for acute coronary syndromes and chronic stable angina [30], [31], [32], [33]. Since renalase specifically metabolizes circulating catecholamines, we sought to determine whether common variation in the renalase gene is associated with inducible ischemia in persons with stable coronary artery disease. Homozygosity for Asp/Asp at codon 37 was associated with inducible wall motion abnormalities during stress echocardiography (OR 1.5) as well as reduced exercise tolerance (multivariable adjusted effect size = −1.0 METS). These findings extend the observation that, in rats, recombinant renalase reduced myocardial infarction size by 54% [10]. Taken together, these data suggest that renalase deficiency or loss of function may contribute to the ischemic cascade in myocardium. Whether renalase replacement therapy can reduce myocardial ischemia in persons with coronary artery disease is not known. We speculate that experimental studies addressing this question may have therapeutic implications in this population.

The main strength of the present study is the collection of detailed phenotypic data derived from comprehensive resting and stress echocardiography in all participants. Combined with the *a priori* selection of the only known common coding polymorphism in renalase, the genotype-phenotype associations reported are biologically plausible and unlikely to represent false positive associations [34]. In addition, confounding by renal impairment is unlikely given the normal mean estimated GFR of study participants. However, several important limitations should be considered in the interpretation of our results. First, our study population was restricted to Caucasian individuals, predominantly men. Therefore, our results may not be generalized to women or to other racial groups, and should be replicated in other populations. Second, we did not measure serum renalase levels or activity as an intermediate phenotype. Finally, our analysis of diastolic dysfunction did not incorporate newer echocardiographic techniques such as tissue Doppler imaging, which would offer further refinement and resolution of phenotype [35].

In conclusion, we found an association of the renalase Glu37Asp polymorphism (C allele) with cardiac hypertrophy, dysfunction, and ischemia in a cross-sectional study of individuals with stable coronary artery disease and normal renal function. The renalase pathway is a novel regulator of circulating catecholamine levels. Future studies should be aimed at determining whether there is a potential therapeutic role for renalase replacement in persons at high risk of cardiovascular morbidity and mortality.
